# Money for operator: the impact of linked agricultural subsidy on incomes

**DOI:** 10.1038/s41598-024-64100-w

**Published:** 2024-06-12

**Authors:** Haoping Yi, Dengwang Guo, Haomin Wang, Guohui Yi, Longzhen Min

**Affiliations:** 1 College of Management, Sichuan Agricultural University, Chengdu, Sichuan China; 2 College of Economics, Sichuan Agricultural University, Chengdu, Sichuan China; 3 Pingxiang Agricultural and Rural Industry Development Service Center, Pingxiang, Jiangxi China

**Keywords:** Socioeconomic scenarios, Statistics

## Abstract

The reform of China’s “three subsidies” has shifted the method of subsidization from payment based on the contracted area to payment based on the actual operational area. Within this context, studying the income-generating impact of the “three subsidies” holds significant practical relevance. Using data from the 2018 China Labor-force Dynamic Survey, this paper employs basic estimation, mediating effect, and moderating effect models to analyze the heterogeneity of agricultural subsidies’ impact on rural household income, the mediating effect of agricultural mechanization, and the moderating effect of operation scale. Our findings indicate that agricultural subsidies, known as the “three subsidies”, have increased total rural household income and agricultural income while decreasing wage income. However, they have shown no significant impact on business income. Notably, agricultural subsidies have significantly elevated the income of food-producing households, with agricultural mechanization partially mediating this effect. Operation scale positively moderates the impact of agricultural subsidies on rural household income and agricultural mechanization. Heterogeneity analysis indicates that agricultural subsidies have a more significant impact on rural household income among agricultural producers in the eastern region.

## Introduction

As agriculture serves as the cornerstone of national economies, issues concerning the enhancement of farmers’ income and agricultural development remain paramount concerns for nations worldwide. Agricultural subsidies stand out as the primary policy tool employed by governments globally to bolster and safeguard agriculture, ensuring food security and fostering growth in farmers’ income^1^. However, the form and extent of subsidies vary among countries and over time.

In terms of the structure of agricultural subsidies, they can be categorized into two main types: decoupled agricultural subsidies and linked agricultural subsidies^2^. There exists a contentious debate regarding the preferable approach to agricultural subsidies. Some scholars argue that decoupled agricultural subsidies offer certain advantages, such as addressing concerns related to obesity and food deserts^3^. However, the prevailing view is that decoupled agricultural subsidies entail the allocation of taxpayers’ funds to agricultural landowners by the government, a practice perceived as an inequitable utilization of public tax revenues that does not directly contribute to societal welfare and public interest^4^. Consequently, linked agricultural subsidies are often regarded as a more favorable approach. Theoretically, linked agricultural subsidies are more policy-driven, as the linkage’s content tends to be closely aligned with the policy objectives of the agricultural subsidy. Agricultural subsidies linked to food production often serve as incentives for agricultural producers to expand their production scale, thereby boosting the country’s food output and productivity^5^. Subsidies linked to ecological protection performance incentivize growers to prioritize ecological considerations in their production practices, fostering environmentally-friendly behaviors that safeguard farmland ecosystems and preserve arable land quality^6^. On the other hand, land-linked agricultural subsidies are aimed at encouraging farmers to cultivate larger areas of land^7^. However, this approach also reinforces the asset value of land and increases the likelihood of agricultural subsidies being absorbed into absolute land rents^8^. While the specific content of agricultural subsidy linkage may align with the country’s current policy objectives, the overarching goal of agricultural subsidies remains to enhance farmers’ incomes and advance agricultural industry development. Despite the significance of linked agricultural subsidies in achieving these objectives, limited attention has been devoted to examining the relationship between such subsidies and income levels.

As an important part of China’s financial support for agriculture, agricultural subsidies have both industrial and income objectives. Through comprehensive and large-scale subsidies, these subsidies aim to promote the development of agricultural industries, narrow the income gap between urban and rural areas, and achieve the coordinated development of industry, agriculture, and urban and rural areas^9^. Since the implementation of China’s agricultural subsidy policy in 2003, the types of subsidies have increased in number and intensity. The traditional method of subsidizing agriculture has come under scrutiny. Despite the cost considerations, the government opted to distribute subsidies based on the contracted land area of farmers. However, this approach has led to an unintended consequence: many farmers who have leased out their land and ceased agricultural production are still eligible to receive agricultural subsidies. In response to this issue, China implemented reforms to the “three subsidies” for agriculture in 2016. Under this new system, subsidies are allocated based on the actual operational area of farmers rather than the contracted land area, effectively linking agricultural subsidies to operation acreage. The question arises: will this revised method of subsidizing farmers effectively enhance their incomes? According to statistics from the Ministry of Agriculture and Rural Affairs, China allocated a total of 143.4 billion yuan in “three subsidies” to aid agricultural production in 2016. Although the relative income gap between urban and rural residents has narrowed in recent years, from 2.99 in 2010 to 2.64 in 2019, the absolute income gap between them has shown an increasing trend, and the effect of agricultural subsidy policies on increasing income is still in doubt.

Many scholars have different views on the expected effect of agricultural subsidies on the increase of farmers’ income^10–12^. Some scholars believe that agricultural subsidies have an income growth effect and can achieve the policy purpose of providing financial support to agriculture^13–18^. However, others make a distinction and question this belief. Gustavo Anríquez found that agricultural subsidies have no impact on promoting farmers’ income^19^. The subsidy funds are small, and the income growth effect of agricultural subsidies is easily offset against the background of falling food prices and increasing production costs. Moreover, agricultural subsidies are eventually converted into land rent, and the increase in land prices offsets the effect of subsidies. Therefore, agricultural subsidies have little or no impact on farmers’ income^20,21^. Some scholars think that due to the continuous emergence of new types of agricultural business entities, there is often a mismatch of resources between the subsidizer and the subsidized party. This mismatch reduces the farmers’ enthusiasm to grow grain and deteriorates grain production and farmers’ income^22^. On the other hand, other scholars believe that, in the initial stage of policy implementation, agricultural subsidies did stimulate farmers’ enthusiasm to grow food, expand the sown area, and further increase food production and farmers’ income. However, in the long run, this effect tends to decline until it disappears^23,24^.

After reviewing existing literature, this paper suggests that the controversy regarding the impact of agricultural subsidies on farmers’ income increase may be due to the heterogeneity of research regions. Moreover, fewer studies have explored the impact path of agricultural subsidies on farmers’ income. Therefore, in order to examine the income-generating effects of the three subsidies, using data from China’s Labor-Force Dynamic Survey (CLDS) in 2018, this paper examines the mediating effect of agricultural mechanization levels in the impact of agricultural subsidies on rural household income and the moderating effect of operation scale on the overall impact path. It also analyzes the income growth effect for individual farmer heterogeneity and regional heterogeneity, drawing on the existing literature. Compared to previous studies, this paper’s contribution is threefold. First, the relationship between agricultural subsidies and rural household income is still contentious, but the conclusion of this study effectively supports the view that agricultural subsidies promote rural household income. Second, the existing literature on agricultural subsidies and farmers’ income increase does not provide a comprehensive analysis of the mechanism of action and the path of influence between the two. This study introduces the mediating variable of agricultural mechanization levels and the moderating variable of operation scale, providing a comprehensive analysis of the mechanisms of the mediating and moderating effects to fill gaps in this regard. Finally, this paper’s sample size includes 3,184 rural households across 29 provinces in China, which lends more credibility to the findings than previous studies with smaller samples.

## Reform process of agricultural subsidies in China

Following the trajectory of China’s agricultural subsidies and building upon the stage division established in existing studies, this paper categorizes the agricultural subsidy-related policies since the founding of New China into four chronological stages.

### Agricultural taxation stage

At this stage, the state has employed various policy instruments to harness the surplus value of agriculture to bolster industrial development^25^. These instruments primarily include:

1. Food purchasing and marketing: This involves the implementation of planned production of agricultural products, limiting the types of agricultural production, state monopoly of agricultural product circulation, and control of sales markets. These measures aim to realize unequal trading to acquire the surplus value of agriculture to support industrial development.
2. Levying agricultural taxes and fees: Agricultural taxes are levied on farmers based on factors such as annual per capita income in rural areas and land differences. The revenue generated is utilized to support industrial development.
3. Adjustment of the price of agricultural means of production: This entails appropriately lowering the price of agricultural means of production to alleviate the impact of agricultural taxes and unified purchasing and marketing on agricultural producers. However, this subsidy is considerably smaller than the surplus value obtained from agriculture at this stage.

### Agricultural tax complementary parallel stage

During this stage, China continued to collect agricultural surplus value through agricultural taxes. However, simultaneous efforts were made to introduce support and subsidy policies aimed at promoting agricultural development^26^. The main policy instruments included:

1. Gradual abolition of the policy of unified purchasing and marketing: This involved restoring the market mechanism for grain and raising grain prices to enable agricultural producers to profit from their produce.
2. Implementation of a special grain reserve system and a policy of protected grain purchase prices: By reforming the grain circulation area and providing protective support for grain purchase prices, these measures aimed to increase agricultural producers' income from grain production.

### Formal agricultural subsidy stage

This stage marks the period when industry supports agriculture, with the state shifting its focus from capturing the surplus value of agriculture to supporting agricultural development through the value generated by secondary and tertiary industries^27^. The main policy instruments include:

1. Gradual abolition of agricultural taxes: Beginning in 2004 and completed by early 2006, this policy entailed the gradual reduction and eventual elimination of agricultural taxes, relieving farmers of the burden of submitting corresponding agricultural taxes and significantly reducing agricultural producers’ financial strain.
2. Subsidies for high-quality seeds: Farmers receive financial subsidies for purchasing superior grain seeds, aimed at reducing production costs and facilitating the adoption of new technologies (such as high-quality seeds). This initiative is designed to improve yields and increase farmers’ incomes.
3. Direct grain subsidies: These subsidies, which have increased in amount and intensity over the years, are provided to producers of staple grain products to incentivize increased grain production.
4. Comprehensive agricultural subsidies: Farmers receive financial subsidies for purchasing agricultural production materials such as seeds, fertilizers, and films. This measure aims to reduce production costs and boost agricultural productivity and income.
5. Subsidies for the purchase of agricultural machinery and equipment: Initially, these subsidies were provided to manufacturers of agricultural machinery and equipment to lower selling prices. Subsequently, the subsidies were redirected to purchasers of agricultural machinery to reduce acquisition costs, thereby promoting the development of agricultural mechanization and enhancing agricultural production levels through subsidy-driven incentives.

### Agricultural subsidy reform stage

In 2015, a pilot agricultural subsidy policy reform was initiated in five provinces, namely Anhui, Shandong, Hunan, Sichuan, and Zhejiang. This reform combined three existing subsidies—comprehensive agricultural subsidies, crop seed subsidies, and direct subsidies for grain planting—into a single subsidy known as the “agricultural support and protection subsidy,” or commonly referred to as the “three subsidy” reform. A significant aspect of this reform was the shift from the previous practice of allocating subsidies based on contracted land area to linking agricultural subsidies with the operational scale of farm households^28^.

Building upon the lessons learned from the pilot phase in 2015, the nationwide implementation of the agricultural subsidy policy reform commenced in 2016, marking a pivotal step in reshaping the agricultural subsidy landscape across the country.

## Materials and methods

### Research hypotheses

#### Direct impact of agricultural subsidy on incomes

Beginning in 2015, the central government initiated a pilot reform of the “three subsidies” in agriculture. This reform consolidated crop seed subsidies, direct subsidies for grain farmers, and comprehensive subsidies for agricultural production materials into agricultural support and protection subsidies. It also explicitly integrated the promotion of appropriate-scale grain operations into its policy objectives^29^. This integration included establishing a separate subsidy fund for appropriate-scale grain operations within the agricultural support and protection subsidy fund, prioritizing support for large-scale farmers in line with the principle of “whoever grows more food will be given priority in support.” In 2016, based on the lessons learned from the pilot phase, the nationwide rollout of the agricultural “three subsidies” reform took place.

Undoubtedly, the alteration in subsidy allocation has led to various changes in agricultural subsidies affecting different types of farm household incomes as follows:

The reform of the “three subsidies” represents a significant shift by linking agricultural subsidies to the actual operational area of farming households^1^. This approach effectively incentivizes grain farmers to expand their agricultural cultivation, thereby boosting their household income from agricultural production^30^. Given China’s prevalence of small-scale farmers and limited per capita arable land, such subsidies play a crucial role in addressing issues like land fragmentation, enhancing the efficiency of agricultural production, and augmenting agricultural income^31^.

However, it’s important to recognize that this shift may also entail a reallocation of time and energy towards family agricultural production, potentially limiting farmers’ engagement in off-farm labor or business ventures, and subsequently impacting wage and business income^32^. Despite these dynamics, the overall impact of the “three subsidies” on total household income remains positive across various household income compositions. For households primarily reliant on agricultural income and engaged in large-scale cultivation, the subsidies serve as a catalyst for increasing both agricultural and total incomes^33^. Conversely, households predominantly dependent on wage or business income may experience less pronounced effects on their labor distribution, especially if they do not engage in land transfers. However, even in cases where agricultural subsidies are indirectly translated into rents, the overall impact on total income remains positive, albeit to a lesser extent^34^. In summary, the “three subsidies” contribute to an overall increase in the total income of rural households, albeit with varying effects on income composition depending on household characteristics and engagement in agricultural activities.Based on this analysis, the following hypotheses (H_1a_, H_1b_, H_1c_, and H_1d_) are formulated:

H_1a_: The “three subsidies” in agriculture contribute to total rural household income.

H_1b_: The “three subsidies” in agriculture contribute to rural household agricultural income.

H_1c_: The “three subsidies” in agriculture suppress rural household wage income.

H_1d_: The “three subsidies” in agriculture suppress rural household business income.

#### The mediation effect of agricultural mechanization level

The “three subsidies” in agriculture, while not directly targeting agricultural machinery, have a profound impact on its adoption and utilization. With the shift towards linking subsidies to the scale of operation, farmers are incentivized to increase their agricultural inputs and focus on production^35^. This heightened investment in other factors of production, such as land, naturally fuels the demand for agricultural mechanization.^36^ Additionally, small-scale farmers are also motivated to invest in agricultural machinery services, thereby facilitating the adoption of mechanization among them^37^.

Overall, the “three subsidies” serve to drive the level of agricultural mechanization among farmers. This, in turn, has a significant impact on farmers’ income. Firstly, improved mechanization enhances agricultural production efficiency, standardizes production processes, and leads to higher yields and better quality agricultural products^38^. This directly contributes to increasing agricultural income.

Furthermore, mechanization facilitates the substitution of manual labor, enabling surplus family labor to be redirected towards off-farm employment or business ventures^39^. This diversification of income sources leads to higher wage and business income for rural families^40^. In essence, the promotion of agricultural mechanization through the “three subsidies” ultimately contributes to the overall increase in total income for rural households.

Based on the above analysis, this paper proposes hypotheses 2a, 2b, 2c, and 2d:

H_2a_: The “three subsidies” in agriculture will contribute to total income by increasing agricultural mechanization level.

H_2b_: The “three subsidies” in agriculture will contribute to agricultural income by increasing agricultural mechanization level.

H_2c_: The “three subsidies” in agriculture will weaken the disincentive effect on wage income by increasing agricultural mechanization level; in other words, agricultural mechanization level has a compensatory effect.

H_2d_: The “three subsidies” in agriculture will weaken the disincentive effect on business income by increasing agricultural mechanization level; in other words, agricultural mechanization level has a compensatory effect.

##### The moderation effect of operation scale

As rural areas in China continue to age and hollow out, land management methods have evolved, and moderate-scale operations with agricultural cooperatives, agricultural machinery cooperatives, and family farms as the mainstay have become the prevailing trend^22^. Due to significant differences in resource endowments among different operators, the same agricultural policy may have varying income effects on different operators^1,42^. Currently, in most regions of China, direct grain subsidies are distributed based on the taxable area of farmers’ operations^43^. Consequently, the size of farmers’ operations determines the amount of their subsidies. Furthermore, in theory, scale operation can enhance resource allocation efficiency, resulting in scale benefits^44^. Farmers operating on a large scale can leverage their factor endowments, information, and social resources to benefit from various agricultural subsidy policies through market-oriented behavior. Building on the aforementioned analysis, this paper proposes hypothesis 3a, 3b, 3c and 3d:

H_3a_: Operation scale inhibits the contribution of the agricultural “three subsidies” to the total income of rural household.

H_3b_: Operation scale inhibits the contribution of the agricultural “three subsidies” to the agricultural income of rural household.

H_3c_: Operation scale will weaken the inhibiting effect of the “three subsidies” on the wage income of rural household.

H_3d_: Operation scale will weakens the inhibiting effect of the “three subsidies” on the business income of rural household.

**Figure 1:**
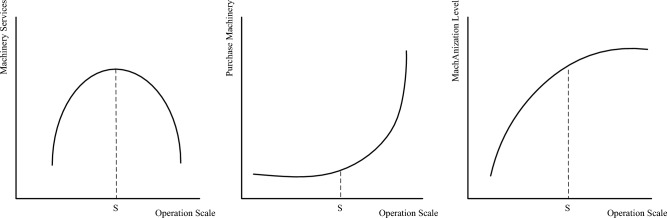
Relationship between operation scale and mechanization level.

Farmers typically achieve agricultural mechanization through outsourced agricultural machinery services or by self-purchasing agricultural machinery. Differences in resource endowments dictate that farmers will adopt different mechanization strategies in conjunction with their agricultural production plans^45^. Some studies have shown an “inverted U-shaped” relationship between operation scale and agricultural machinery outsourcing services, as illustrated in Fig. 1. Before the threshold value S, the expansion of operation scale increases the demand for agricultural machinery outsourcing services^46^. Meanwhile, the demand for self-purchased agricultural machinery is not significant, and thus the stimulating effect of agricultural subsidies on farmers’ self-purchased agricultural machinery is not obvious at this stage. However, after the threshold value S, as depicted in Fig. 1, the cost of self-purchased agricultural machinery is lower than the cost of outsourced agricultural machinery services. As a result, farmers tend to reduce the purchase of agricultural machinery services and increase the demand for self-purchased agricultural machinery^47^. As the scale of agricultural operations increases, farmers tend to enhance their mechanization levels^48^. This dynamic weakens the impact of agricultural subsidies on driving farm mechanization levels higher, indicating a substitution relationship between operation scale and agricultural subsidies in terms of mechanization level enhancement. Larger-scale farmers typically already operate with higher mechanization levels, leading them to allocate more agricultural subsidies towards other aspects of their operations.

**Figure 2:**
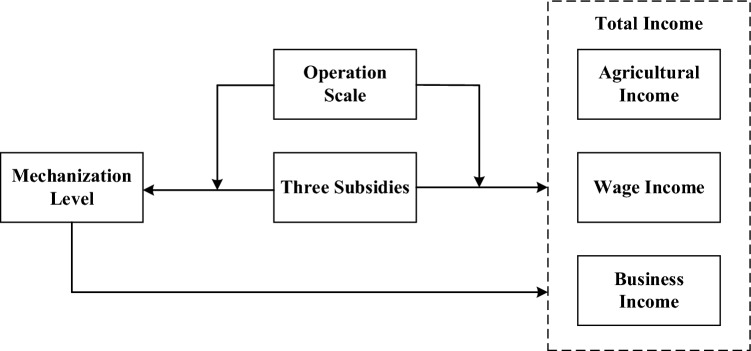
The logical framework for agricultural subsidy to affect incomes.

Based on the above analysis, this paper proposes hypothesis 4:

H_4_: Operation scale has a negative moderating effect on the relationship between agricultural subsidies and agricultural mechanization levels. The logical framework of this paper is shown in Fig. 2.

### Data source

The data used in this study are mainly from the China Labor-force Dynamic Survey (CLDS) conducted by Sun Yat-sen University in 2018. It contains tracking and cross-sectional data at three levels: individual labor force, household, and community. The CLDS is a large-scale interdisciplinary tracking survey that targets the working-age population aged 15–64. It focuses on the current situation and changes in the labor force’s education, employment, labor rights, occupational mobility, occupational protection and health, occupational satisfaction and well-being, as well as the political, economic, and social development of the labor force’s community, demographic structure, household property and income, household consumption, household giving, rural household production, land, and many other topics of the labor force’s household. In this study, only rural household income is discussed, and only the rural sample is kept. After dealing with missing values and outliers, a total of 3,184 rural households’ data are studied in this paper.

## Methods

### Basic estimation model

To examine the impact of agricultural subsidies on rural household income, the following basic estimation model is constructed:

1 $${\varvec{l}}{\varvec{n}}{{\varvec{I}}{\varvec{n}}{\varvec{c}}}_{{\varvec{i}}}={\alpha }_{0}+{\alpha }_{1}{{\varvec{S}}{\varvec{u}}{\varvec{b}}}_{{\varvec{i}}}+{\alpha }_{2}{{\varvec{C}}{\varvec{o}}{\varvec{n}}}_{{\varvec{i}}}+{\varepsilon }_{i}$$

In Eq. (1), ${{\varvec{I}}{\varvec{n}}{\varvec{c}}}_{{\varvec{i}}}$ represents the income of rural household $i$ in 2017, include total income, agricultural incme, wage income and business incme; ${\alpha }_{0}$ is a constant term; ${{\varvec{S}}{\varvec{u}}{\varvec{b}}}_{{\varvec{i}}}$ represents the total agricultural subsidies received by household $i$ in 2017; ${\alpha }_{1}$ represents the regression coefficient, which measures the effect of agricultural subsidies on rural household income; ${{\varvec{C}}{\varvec{o}}{\varvec{n}}}_{{\varvec{i}}}$ represents the control variables; ${\alpha }_{2}$ represents the regression coefficient of control variables, which measures the effect of other control variables on rural household income; ${\varepsilon }_{i}$ represents the random disturbance term.

### Mediation effect model

In this paper, when analyzing the effect of agricultural subsidies on rural household income, if agricultural subsidies affect rural household income through the mediation variable agricultural mechanization level, a mediation effect analysis is required. There are various methods for mediation effect analysis, and the more common methods include causal steps approach, Sobel test and Bootstrap test^49,50^. Referring to the study by Baron and Kenny50, this paper applies the causal steps approach to test the mediation effect of agricultural mechanization level, and its mediation effect model is as follows.

2 $${{\varvec{l}}{\varvec{n}}{\varvec{I}}{\varvec{n}}{\varvec{c}}}_{{\varvec{i}}}=c{{\varvec{S}}{\varvec{u}}{\varvec{b}}}_{{\varvec{i}}}+{\mu }_{1}$$

3 $${{\varvec{M}}{\varvec{e}}{\varvec{d}}}_{{\varvec{i}}}=a{{\varvec{S}}{\varvec{u}}{\varvec{b}}}_{{\varvec{i}}}+{\mu }_{2}$$

4 $${{\varvec{l}}{\varvec{n}}{\varvec{I}}{\varvec{n}}{\varvec{c}}}_{{\varvec{i}}}=c^{\prime}{{\varvec{S}}{\varvec{u}}{\varvec{b}}}_{{\varvec{i}}}+b{{\varvec{M}}{\varvec{e}}{\varvec{d}}}_{{\varvec{i}}}+{\mu }_{3}$$

In Eqs. (2) to (4), ${{\varvec{I}}{\varvec{n}}{\varvec{c}}}_{{\varvec{i}}}$ represents the total income of rural household $i$ in 2017; ${{\varvec{S}}{\varvec{u}}{\varvec{b}}}_{{\varvec{i}}}$ represents the total agricultural subsidies received by household $i$ in 2017; ${{\varvec{M}}{\varvec{e}}{\varvec{d}}}_{{\varvec{i}}}$ is the mediating variable, representing the operation scale of farm household $i$ in 2018; ${\mu }_{1}$、${\mu }_{2}$、${\mu }_{3}$ represent the random disturbance terms.

### Mediated moderation model

After the mediating effect test, adding moderation variables needs to consider the moderation effect. Drawing on the research of Baron and Edwards^51,52^, a regression model with a product term is used to test and construct the following equation to test the moderating effect model of operation scale:

5 $${\varvec{l}}{\varvec{n}}{\varvec{I}}{\varvec{n}}{\varvec{c}}={\beta }_{0}+{\beta }_{1}{\varvec{S}}{\varvec{u}}{\varvec{b}}+{\beta }_{2}{\varvec{M}}{\varvec{o}}{\varvec{d}}+{\beta }_{3}{\varvec{S}}{\varvec{u}}{\varvec{b}}\times {\varvec{M}}{\varvec{o}}{\varvec{d}}+{\beta }_{4}{\varvec{C}}{\varvec{o}}{\varvec{n}}+{\theta }_{1}$$

6 $${\varvec{M}}{\varvec{e}}{\varvec{d}}={\gamma }_{0}+{\gamma }_{1}{\varvec{S}}{\varvec{u}}{\varvec{b}}{\varvec{s}}+{\gamma }_{2}{\varvec{M}}{\varvec{o}}{\varvec{d}}+{\gamma }_{3}{\varvec{S}}{\varvec{u}}{\varvec{b}}\times {\varvec{M}}{\varvec{o}}{\varvec{d}}+{\gamma }_{4}{\varvec{C}}{\varvec{o}}{\varvec{n}}+{\theta }_{2}$$

7 $${\varvec{l}}{\varvec{n}}{\varvec{I}}{\varvec{n}}{\varvec{c}}={\delta }_{0}+{\delta }_{1}{\varvec{S}}{\varvec{u}}{\varvec{b}}+{\delta }_{2}{\varvec{M}}{\varvec{o}}{\varvec{d}}+{\delta }_{3}{\varvec{S}}{\varvec{u}}{\varvec{b}}\times {\varvec{M}}{\varvec{o}}{\varvec{d}}+{\delta }_{4}{\varvec{M}}{\varvec{e}}{\varvec{d}}+{\delta }_{5}{\varvec{M}}{\varvec{e}}{\varvec{d}}\times {\varvec{M}}{\varvec{o}}{\varvec{d}}+{\delta }_{6}{\varvec{C}}{\varvec{o}}{\varvec{n}}+{\theta }_{3}$$

In Eqs. (5) to (7), ${\varvec{M}}{\varvec{o}}{\varvec{d}}$ represents the moderation variable, ${\varvec{S}}{\varvec{u}}{\varvec{b}}\times {\varvec{M}}{\varvec{o}}{\varvec{d}}$ represents the product term of the independent variable and the moderation variable, and ${\varvec{M}}{\varvec{e}}{\varvec{d}}\times {\varvec{M}}{\varvec{o}}{\varvec{d}}$ represents the product term of the mediation variable and the moderation variable. Equations (5) to (7) can be used to distinguish whether the moderation variable moderates the direct path, the first half path, and the second half path of the mediation process, and the coefficient of ${\varvec{S}}{\varvec{u}}{\varvec{b}}\times {\varvec{M}}{\varvec{o}}{\varvec{d}}$ is the moderation effect of the moderation variable on each path: When ${\beta }_{3}$ is significant in Eq. (5), operation scale moderates the direct path; When ${\gamma }_{3}$ in Eq. (6) is significant and ${\delta }_{4}$ in Eq. (7) is significant, operation scale moderates the front radius; when ${\gamma }_{1}$ inequation (6) is significant and ${\delta }_{5}$ in Eq. (7) is significant, operation scale moderates the back radius; when ${\gamma }_{3}$ in Eq. (6) is significant and ${\delta }_{5}$ in Eq. (7) is significant, operation scale moderates the front and back radius.

### Variable selection

1. Dependent variable. Rural household income comprises total income, agricultural income, wage income, and business income. Given the considerable disparities in total income across different rural households, we applied a natural logarithmic function to transform the total income data. This transformation mitigates the discrepancies between the highest and lowest income levels, allowing us to utilize the ln-transformed total income as the dependent variable to represent the income level of rural households.
2. Independent variables. The “three subsidies” in agriculture, also referred to as agricultural support and protection subsidies, were consolidated from crop seed subsidies, direct subsidies for grain farmers, and comprehensive subsidies for agricultural production materials.
3. Mediating variable. To examine the mediating effect of agricultural mechanization level on the impact of agricultural subsidies on rural household income, we used the following questionnaire: “What is the farming method for food crop production in your household?” The level of agricultural mechanization was classified into three categories based on the responses: “fully mechanized”, “partially mechanized” and “traditional farming”.
4. Moderating variable. In order to examine the moderating effect of operation scale on the relationship between agricultural subsidies and rural household income, we used the following questionnaire: “How many m^2^ of land did your family have last year?” We used the area of arable land as the operation scale.
5. Instrumental variables. To account for self-selection bias and endogeneity problems resulting from reverse causality, we used the instrumental variables method. We selected the “three subsidies” standard of village residence as the instrumental variables of agricultural subsidy. Additionally, we selected the terrain as the instrumental variable of agricultural mechanization level to eliminate endogeneity.
6. Control Variables. There are many factors that can affect rural household income. In order to control for the heterogeneity among individual farmers and to follow the principle that control variables should be as exogenous as possible, this paper includes multiple levels of control variables, such as gender, age, marital status, production training, paved roads, presence of a village bus station, village location, households with professional occupations, availability of piped water in the village, the presence of a farmers’ market in the village, and needy family.

Table 1 displays the descriptive statistics of each variable. The average total income of farm families in the sample was 37,211 yuan, with an average farm income of 16,662 yuan, an average wage income of 17,283 yuan, and an average business income of 4,382 yuan. Notably, there is a considerable standard deviation in both total income and operating income, indicating significant variation in income levels among different households. On average, farm households received 1,135 yuan in agricultural subsidies. The average farm size was 11.019 mu, with agricultural mechanization predominantly at a partial level. The average standard of agricultural subsidies across villages was 907 yuan. Most villages were characterized by mountainous and plains terrain. Regarding demographics, 48.9% of the farmers interviewed were male, 88.2% were married, and the average age ranged from 53 to 54 years. Around 72.6% of villages provided agricultural production training, 93.2% had paved roads, and 88.5% had access to piped water. However, only 35.2% of villages had a public transportation station, 18.2% had a farmers' market, and 4.1% were located in peri-urban areas. In addition, 45.6% of families were recognized as needy.

**Table 1:** Definition and descriptive statistics of each variable.

Variables	Definition	Mean	SD^a^	N^b^
Total Income	Total income of rural household (thousand yuan)	37.211	54.143	3,071
Agricultural Income	Agricultural production income of rural household (thousand yuan)	16.662	2.717	3,182
Wage Income	Wage income of rural household (thousand yuan)	17.283	3.540	3,184
Business Income	Business income of rural household (thousand yuan)	4.382	38.908	3,183
Agricultural Subsidy	Three-agricultural subsidy received by rural household (thousand yuan)	1.135	3.517	3,184
Operation Scale	The area of farmland operated by rural household (mu)	11.019	24.613	3,162
Mechanization Level	Machinery model (1 = Traditional farming; 2 = Partial Mechanization; 3 = Full Mechanization)	2.023	0.725	3,020
Village Standard of Subsidy	The standard of subsidy for grain farmers in different villages (thousand yuan)	0.907	2.199	3,155
Terrain	Village Terrain (1 = Mountainous; 2 = Hilly; 3 = Plain)	2.319	0.841	3,184
Gender	Gender of the household head (0 = Female; 1 = Male)	0.489	0.500	3,184
Age	The age of household head (age)	53.571	93.268	3,184
Marital status	The marital status of household head(0 = Unmarried; 1 = Married)	0.882	0.323	3,184
Production Training	Whether villages organize agricultural production training (0 = No; 1 = Yes)	0.726	0.446	3,184
Hardening Pavement	Whether the road of village is hardened (0 = No; 1 = Yes)	0.932	0.252	3,184
Village Bus Station	Availability of bus stops in the village (0 = No; 1 = Yes)	0.352	0.478	3,184
Village Location	Whether the village is located in a peri-urban area (0 = No; 1 = Yes)	0.041	0.198	3,184
Professional Households	Whether the household is specialized in agricultural production (0 = No; 1 = Yes)	0.102	0.303	3,184
Piped Water in the Village	Availability of piped water in the village (0 = No; 1 = Yes)	0.885	0.319	3,184
Village Farmers’ Market	Availability of farmers' markets in the village (0 = No; 1 = Yes)	0.182	0.386	3,184
Needy Family	Whether the family is needy (0 = No, 1 = Yes)	0.456	0.498	3,184

^a^SD = Standard deviation; ^b^Number of observations.

## Results

Before conducting the empirical analysis, it is necessary to test for the presence of multicollinearity between the variables. Agricultural subsidiy and agricultural mechanization level are positively correlated with total income, agricultural income and wage income at the 1% level of significance, and agricultural subsidies are positively correlated with the level of agricultural mechanization at the 1% level of significance. The maximum variance inflation factor (VIF) is 1.17, which indicates that there is no multicollinearity problem among the variables used in this paper.

### Model results

Table 2 presents the results of the baseline regressions, illustrating the effects of the three agricultural subsidies on total rural household income, agricultural income, wage income, and business income, with and without control variables. Specifically, in rows (1)–(4) of Table 2, the impacts of agricultural subsidies on both total income and agricultural income are significantly positive at the 1% confidence level. This indicates that agricultural subsidies contribute positively to the total income of rural households as well as their agricultural income, confirming Hypotheses 1a and 1b. Moreover, in columns (5)–(8) of Table 2, the impact of agricultural subsidies on wage income and business income is negative. The impact on wage income is significant at the 1% confidence level, while the impact on business income is not significant. This validates Hypothesis 1c but does not support Hypothesis 1d. This discrepancy could be attributed to the relatively small proportion of business income in rural household income, as most rural households are not engaged in business activities, leading to the insignificant result for business income. This analysis suggests that agricultural subsidies play a significant role in boosting total income and agricultural income for rural households, although their impact on wage income is negative, possibly due to the limited contribution of business income to overall household income in rural areas.

**Table 2:** Impact of agricultural subsidies on rural household income.

Variables	Total income		Agricultural income		Wage income		Business income	
	OLS		OLS		OLS		OLS	
	(1)	(2)	(3)	(4)	(5)	(6)	(7)	(8)
Agricultural subsidy	0.089***	0.073***	0.608***	0.591***	− 0.511***	− 0.471***	− 0.050	− 0.058
	(0.015)	(0.015)	(0.049)	(0.049)	(0.069)	(0.069)	(0.041)	(0.042)
Gender		− 0.076**		0.039		− 0.372**		0.036
		(0.037)		(0.125)		(0.175)		(0.106)
Age		− 0.0002		0.0005		0.001		− 0.001
		(0.0002)		(0.001)		(0.001)		(0.001)
Marital status		0.015		0.341*		− 0.252		− 0.138
		(0.058)		(0.194)		(0.273)		(0.165)
Production Training		− 0.130***		0.100		0.290		− 0.100
		(0.042)		(0.142)		(0.201)		(0.121)
Hardening Pavement		0.048		− 1.796***		0.701*		0.503**
		(0.077)		(0.260)		(0.367)		(0.222)
Village Bus Station		0.189***		− 0.015		− 0.014		0.243**
		(0.040)		(0.135)		(0.190)		(0.115)
Village Location		0.110		0.443		− 0.498		0.730**
		(0.098)		(0.337)		(0.474)		(0.287)
Professional Households		− 0.013		0.556***		− 1.442***		0.204
		(0.062)		(0.210)		(0.296)		(0.179)
Piped Water in the Village		0.067		2.493***		0.206		0.399**
		(0.065)		(0.210)		(0.295)		(0.179)
Village Farmers’ Market		0.067		0.198		0.272		0.343**
		(0.050)		(0.172)		(0.243)		(0.147)
Needy Family		− 0.791***		0.342***		− 1.566***		− 0.384***
		(0.0368)		(0.125)		(0.176)		(0.107)
Constant	9.456***	9.870***	3.805***	2.696***	7.376***	7.291***	1.303***	0.710*
	(0.097)	(0.141)	(0.311)	(0.473)	(0.433)	(0.667)	(0.259)	(0.404)
Observations	3,071	3,071	3,182	3,182	3,184	3,184	3,183	3,183
R^2^	0.011	0.153	0.045	0.103	0.017	0.054	0.001	0.019

*, **, *** indicate 10%, 5% and 1% significant levels, respectively.

### Robustness analysis

In this paper, we conducted robustness checks by adding control variables and employing the Tobit model to validate the benchmark regression results. Notably, the Tobit model employed in this study incorporates a two-way subsumption with 5% left subsumption and 95% right subsumption, which helps prevent abnormally large and small values from affecting the analysis. Table 3 presents the endogeneity test results, where the impact of agricultural subsidies on total rural household income and agricultural income is significantly positive at the 1% confidence level. However, the impact on wage income is significantly negative at the 1% confidence level, while the impact on business income remains nonsignificant. These findings are consistent with the results observed in Table 2, indicating the robustness of our results. Overall, the inclusion of control variables and the utilization of the Tobit model strengthen the reliability of our regression analysis, demonstrating consistent and robust results regarding the effects of agricultural subsidies on various income components of rural households.

**Table 3:** Robustness analysis results.

Variables	Total income		Agricultural income		Wage income		Business income	
	Tobit		Tobit		Tobit		Tobit	
	(1)	(2)	(3)	(4)	(5)	(6)	(7)	(8)
Agricultural Subsidy	0.312***	0.328***	1.628***	1.619***	− 12.813***	− 11.576***	− 3.038	− 3.894
	(0.061)	(0.062)	(0.157)	(0.155)	(2.593)	(2.467)	(2.257)	(2.325)
Constant	9.409***	10.096***	2.260***	1.004***	61.390	57.138	− 93.324***	− 136.255**
	(0.357)	(0.642)	(0.875)	(1.382)	(13.700)	(18.724)	(24.839)	(38.629)
Control Variables	No	Yes	No	No	No	No	No	Yes
Observations	3,071	3,071	3,182	3,182	3,184	3,184	3,183	3,183
Pseudo R^2^	0.026	0.081	0.022	0.052	0.011	0.034	0.001	0.026

*, **, *** indicate 10%, 5% and 1% significant levels, respectively.

### Endogeneity analysis

The core independent variable of this paper is agricultural subsidies, and the mediating variable is agricultural mechanization level. However, these variables may have self-selection bias and reverse causality on rural household income. To address this endogeneity issue, this paper adopts an instrumental variable method. Many scholars have used regional-level agricultural subsidy standards such as municipal agricultural subsidy standards as instrumental variables for agricultural subsidies^53^. The reason is that regional agricultural subsidy standards have a significant positive effect on the agricultural subsidies received by farmers in the region, satisfying the requirement of correlation. At the same time, regional subsidy standards only have an effect on the subsidies received by farmers, satisfying the requirement of exogeneity. Similarly, the larger standard of subsidies in villages, the more subsidies farmers will receive, while it has no direct effect on rural household income. Therefore, in this paper, the standard of subsidies in village is used as instrumental variables for agricultural subsidies. For agricultural mechanization level, Duflo and Pand used the average slope of the county as an instrumental variable^54^. In this paper, village topography is used as the instrumental variable because it is a natural geographical factor that only affects rural household income through agricultural mechanization level, satisfying the requirements of correlation and exogeneity.

This paper selects the two-stage least squares method to regress the instrumental variables with robust standard errors. Table 4 displays the instrumental variable regression for agricultural subsidies. In columns (1)-(3), the effects of agricultural subsidy standards in villages on agricultural subsidies are all significant at the 1% confidence level. This indicates that the instrumental variable is effective in explaining endogenous explanatory variables. Furthermore, the F-statistics for the significance of the coefficients of the instrumental variables in the one-stage regression are 59.93, 60.64, and 60.66, respectively, surpassing the standard value of 10, which indicates the absence of weak instrumental variables. In the two-stage regression results, agricultural subsidies significantly affect total income at the 5% confidence level, and agricultural income and wage income at the 1% confidence level. Comparing these results with the benchmark regression in Table 2, the coefficients of agricultural subsidies on total income, agricultural income, and wage income change from 0.088, 0.585, and − 0.443 to 0.074, 1.116, and − 0.883, respectively. This suggests that due to the endogeneity issue, the boosting effect of agricultural subsidies on total income is overestimated, while the boosting effect on agricultural income is underestimated. Additionally, it underestimates the dampening effect of agricultural subsidies on wage income. Overall, the instrumental variable regression results provide further insight into the impact of agricultural subsidies on different income components, accounting for endogeneity concerns and offering adjusted coefficients that more accurately reflect the true relationship.

**Table 4:** Endogeneity analysis results of agricultural subsidy.

Variables	2SLS (Phase I)			2SLS (Phase II)		
	Agricultural subsidy			Total income	Agricultural income	Wage income
	(1)	(2)	(3)	(4)	(5)	(6)
Agricultural subsidy				0.074**	1.116***	− 0.883***
				(0.029)	(0.110)	(0.140)
Standard of the subsidy	0.320***	0.314***	0.314***			
	(0.014)	(0.015)	(0.014)			
Constant	4.562***	4.504***	4.504***	9.871***	− 0.703	9.98***
	(0.142)	(0.147)	(0.147)	(0.219)	(0.801)	(1.040)
Control variables	Yes	Yes	Yes	Yes	Yes	Yes
Observations	3,042	3,153	3,155	3,042	3,153	3,155
R^2^	0.302	0.288	0.288	0.153	0.070	0.043
Phase I F-value	59.93	60.64	60.66	\	\	\

*, **, *** indicate 10%, 5% and 1% significant levels, respectively.

In Table 5, the instrumental variable regression for the level of agricultural mechanization is presented. Columns (1) to (3) demonstrate that the effect of the village's terrain on the level of agricultural mechanization is significant at the 1% confidence interval. This indicates that the instrumental variable effectively explains endogenous explanatory variables. Moreover, the F-statistics for the significance of the coefficients of instrumental variables in the first-stage regression are 65.15, 63.82, and 63.82, respectively, exceeding the standard value of 10. This suggests the absence of weak instrumental variables. In the two-stage regression results, mechanization level significantly impacts total income and agricultural income at the 1% confidence interval, and significantly impacts wage income at the 5% confidence interval.

**Table 5:** Endogeneity analysis results of mechanization level.

Variables	2SLS (Phase I)			2SLS (Phase II)		
	Agricultural subsidy			Total income	Agricultural income	Wage income
	(1)	(2)	(3)	(4)	(5)	(6)
Mechanization Level				0.465***	1.534***	0.834**
				(0.070)	(0.237)	(0.326)
Terrain	0.360***	0.346***	0.346***			
	(0.015)	(0.014)	(0.144)			
Constant	0.970***	1.042***	1.043***	9.473***	3.651***	2.636***
	(0.081)	(0.078)	(0.078)	(0.182)	(0.588)	(0.815)
Control Variables	Yes	Yes	Yes	Yes	Yes	Yes
Observations	2,920	3,018	3,020	2,920	3,018	3,020
R^2^	0.195	0.183	0.183	0.102	0.025	0.026
Phase I F-value	65.15	63.82	63.82	\	\	\

*, **, *** indicate 10%, 5% and 1% significant levels, respectively.

### Heterogeneity analysis

There is a high degree of diversity in China’s natural and socioeconomic conditions, and the individual resources and decision-making behaviors of farmers also vary^55^. Agricultural subsidies, as a policy of financial support for agriculture, are closely related to local financial levels and regional natural endowments, and there are differences in the standards of agricultural subsidies in different regions. Similar to Ben-David’s study of other countries, China has gradually formed identifiable regions in the east, central, and west since its reform and opening up, and has developed a trend of internal convergence^[5656]^. To examine the heterogeneity of income among food-producing households, we have categorized China’s economic regions into three main areas: eastern, central, and western regions, following the official division by the National Bureau of Statistics of China. Eastern China comprises the following provinces and municipalities: Liaoning, Jilin, Heilongjiang, Beijing, Tianjin, Hebei, Shanghai, Jiangsu, Zhejiang, Fujian, Shandong, Guangdong, and Hainan. The central region includes: Shanxi, Anhui, Jiangxi, Henan, Hubei, and Hunan. The western region encompasses: Inner Mongolia, Guangxi, Chongqing, Sichuan, Guizhou, Yunnan, Tibet, Shaanxi, Gansu, Qinghai, Ningxia, and Xinjiang. We continued our analysis using model (1), and the specific results are shown in Table 6.

**Table 6:** Regional heterogeneity analysis results.

Variables	Total income			Agricultural income			Wage income		
	Eastern	Central	Western	Eastern	Central	Western	Eastern	Central	Western
	(1)	(2)	(3)	(4)	(5)	(6)	(7)	(8)	(9)
Agricultural Subsidy	0.084***	0.061	0.011	1.002***	0.742***	0.076	− 0.625***	− 0.845***	− 0.281**
	(0.019)	(0.043)	(0.027)	(0.071)	(0.141)	(0.076)	(0.102)	(0.186)	(0.124)
Constant	9.735***	10.312***	10.476***	2.165***	1.426	5.324***	7.211***	9.572***	8.684***
	(0.201)	(0.320)	(0.366)	(0.772)	(1.070)	(0.988)	(1.104)	(1.406)	(1.608)
Control Variables	Yes	Yes	Yes	Yes	Yes	Yes	Yes	Yes	Yes
Observations	1,285	895	891	1,298	974	910	1,300	974	910
R-squared	0.187	0.179	0.186	0.159	0.138	0.081	0.089	0.063	0.078

*, **, *** indicate 10%, 5% and 1% significant levels, respectively.

In terms of total income, agricultural subsidies exhibit a significant positive effect on total income exclusively in the eastern region, whereas no such effect is observed in the central and western regions. This discrepancy can be attributed to the varying levels of regional finance, with the eastern region, being more developed, benefitting from better local financial resources, thus experiencing a significant impact. In terms of agricultural income, the influence of agricultural subsidies on agricultural income is significantly positive in both the eastern and central regions, with a greater effect observed in the eastern region compared to the central region. However, no significant impact is observed in the western region. This disparity is likely due to the concentration of major grain-producing provinces in the eastern and central regions, while the western region, except for Sichuan Province, lacks significant grain production. Additionally, the economic development gap and inadequate local finances in the western region contribute to the limited impact of agricultural subsidies on income enhancement. From the perspective of wage income, agricultural subsidies demonstrate a significant negative impact on wage income across all three regions, with the central region experiencing the most substantial impact, followed by the eastern and western regions. This finding aligns with data from the 2022 National Bureau of Statistics, indicating that the central region has a significant share of cross-provincial migrant workers, amplifying the impact of agricultural subsidies on wage income in this region. In conclusion, the regional disparities in the impact of agricultural subsidies.

### Mediation effect analysis

Table 7 presents the results of the mediation effect analysis of agricultural mechanization levels. It has already been concluded that agricultural subsidies have a statistically significant positive impact on rural household income at the 1% level in Table 2. Columns (1) of Table 7 demonstrate that agricultural subsidies also have a statistically significant positive effect on agricultural mechanization levels at the 1% level. Table 7 illustrates that both agricultural subsidies and the level of mechanization significantly influence all income categories, indicating a mediating effect of agricultural mechanization. Specifically, both agricultural subsidies and mechanization level positively impact total income and agricultural income, affirming Hypotheses 2a and 2b. This suggests that agricultural subsidies contribute to the increase in rural households’ total income and agricultural income by enhancing the level of mechanization. Moreover, agricultural subsidies have a negative effect on wage incomes, whereas the level of mechanization has a positive effect. This indicates that agricultural subsidies mitigate the inhibitory effect on wage incomes by promoting mechanization, demonstrating a masking effect of agricultural mechanization. Hypothesis 2c is supported by these findings. Overall, these results underscore the intricate relationship between agricultural subsidies, mechanization level, and various income components, highlighting the importance of mechanization in moderating the impact of agricultural subsidies on rural household incomes.

**Table 7:** Mediation effect analysis results.

Variables	Mechanization level	Total income	Agricultural income	Wage income
	OLS	OLS	OLS	OLS
	(1)	(2)	(3)	(4)
Agricultural Subsidy	0.133***	0.062***	0.570***	− 0.485***
	(0.010)	(0.015)	(0.051)	(0.073)
Mechanization Level		0.093***	0.212**	0.222**
		(0.027)	(0.091)	(0.130)
Constant	1.118***	9.804***	2.575***	6.983***
	(0.095)	(0.147)	(0.487)	(0.693)
Control Variables	Yes	Yes	Yes	Yes
Observations	3,020	2,920	3,018	3,020
R^2^	0.091	0.157	0.106	0.053

*, **, *** indicate 10%, 5% and 1% significant levels, respectively.

### Mediated moderation effect analysis

Next, we explore the moderation effect of operation scale on the impact of agricultural subsidy on rural household income. As presented in column (1)-(3) of Table 8, the coefficient of the interaction term between agricultural subsidy and operation scale is significant. This indicates that operation scale has a moderation effect on the direct effect, and hypothesis 3a, 3b and 3c is confirmed. As shown in columns (4)-(7)) of Table 8, the coefficients of the interaction term between agricultural subsidy and operation scale are significant, indicating that there is a moderation effect of operation scale on the front radius of the mediation effect, and hypothesis 4 is confirmed. However, the coefficient of the interaction term between agricultural mechanization level and operation scale in column (5)-(7) is not significant, suggesting that agricultural mechanization level does not have a moderation effect on the back radius of the mediation effect. To improve farmers’ income and promote rural revitalization and common prosperity, it is not only necessary to increase the strength of agricultural subsidies but also to improve the precision of agricultural subsidies, guide farmers to operate at an appropriate scale, cultivate new agricultural business entities, and enhance the policy effect of agricultural subsidy.

**Table 8:** Mediated moderation effect analysis results.

Variables	Total income	Agricultural income	Wage income	Mechanization level	Total income	Agricultural income	Wage income
	OLS	OLS	OLS	OLS	OLS	OLS	OLS
	(1)	(2)	(3)	(4)	(5)	(6)	(7)
Agricultural Subsidy	0.076***	0.659***	− 0.491***	0.147***	0.065***	0.642***	− 0.501***
	(0.016)	(0.054)	(0.076)	(0.011)	(0.017)	(0.057)	(0.081)
Operation Scale	0.022***	0.121***	− 0.148***	0.006**	0.024***	0.117***	− 0.123***
	(0.004)	(0.014)	(0.020)	(0.003)	(0.006)	(0.020)	(0.028)
Mechanization Level					0.099***	0.156	0.353**
					(0.033)	(0.110)	(0.157)
Subsidy × Operation Scale	− 0.002***	− 0.012***	0.014***	− 0.001**	− 0.002***	− 0.012***	0.014***
	(0.0005)	(0.002)	(0.002)	(0.0003)	(0.0005)	(0.002)	(0.002)
Mechanization Level × Operation Scale					− 0.001	0.001	− 0.012
					(0.002)	(0.007)	(0.010)
Constant	9.813***	2.088***	7.800***	1.032***	9.729***	2.060***	7.195***
	(0.147)	(0.490)	(0.692)	(0.100)	(0.162)	(0.533)	(0.758)
Control Variables	Yes	Yes	Yes	Yes	Yes	Yes	Yes
Observations	3,049	3,160	3,162	3,002	2,902	3,000	3,002
R^2^	0.163	0.124	0.075	0.094	0.165	0.127	0.074

*, **, *** indicate 10%, 5% and 1% significant levels, respectively.

## Conclusions and discussions

Based on data from the 2018 China Labor-Force Dynamics Survey, this paper discusses the impact of agricultural subsidies on rural household income and introduces agricultural mechanization level and operation scale to analyze their specific role in the impact mechanism. The findings show that: First, the agricultural “three subsidies” significantly contribute to total rural household income. Specifically, they have a notable positive impact on agricultural income, a significant negative effect on wage income, and no significant effect on business income. Secondly, agricultural subsidies, known as the “three subsidies”, promote the overall increase in total income and agricultural income of rural households by boosting the level of mechanization. Additionally, they alleviate the negative impact of subsidies on wage income by increasing mechanization levels. Third, the impact of the agricultural “three subsidies” on income weakens as the scale of farm operations increases. Similarly, the effect of agricultural mechanization on income also diminishes with larger farm sizes. Fourth, heterogeneity analysis reveals regional differences, with the impact of agricultural subsidies on income gradually decreasing from the eastern region to the central and western regions. Combining the findings of this paper, the following policy implications are obtained:

Firstly, in summary, the three agricultural subsidies have indeed led to an increase in the total income of rural households. It is imperative to continue these subsidies as they play a pivotal role in encouraging farmers to cultivate and augment their incomes. Secondly, it is crucial to note the dampening effect of agricultural subsidies on wage incomes. This policy effect indicates that farmers are directing more efforts towards agricultural production. Therefore, the current agricultural subsidies, structured based on the actual operational area, align well with the initial intent of the policy. Thirdly, regional heterogeneity plays a significant role in the growth effect of agricultural subsidies on rural household income. Hence, it is crucial for the country to tailor its agricultural subsidy policies according to regional resource endowments and economic development conditions. Agricultural subsidies exhibit a substantial growth effect on rural household income in the eastern region. However, for the central and western regions, the standard of agricultural subsidies tends to be lower due to the varying levels of regional economic development and the financial capacities of local governments. Consequently, the impact of agricultural subsidies on income growth is less pronounced, primarily due to the significant number of laborers migrating out of these regions. Nevertheless, given that the central and western regions are traditional agricultural areas in China with a considerable demand for agricultural support, it is imperative to consider raising the subsidy standards in these regions to effectively stimulate income growth and support agricultural development.

### Supplementary Information

Supplementary Information.

## Data Availability

All data generated or analysed during this study are included in this published article and its supplementary information files.
